# The Influence of Lyophilized EmuGel Silica Microspheres on the Physicomechanical Properties, *In Vitro* Bioactivity and Biodegradation of a Novel Ciprofloxacin-Loaded PCL/PAA Scaffold

**DOI:** 10.3390/polym8060232

**Published:** 2016-06-15

**Authors:** Mostafa Mabrouk, Yahya Essop Choonara, Pradeep Kumar, Lisa Claire du Toit, Viness Pillay

**Affiliations:** 1 Wits Advanced Drug Delivery Platform Research Unit, Department of Pharmacy and Pharmacology, School of Therapeutics, Faculty of Health Sciences, University of the Witwatersrand, Johannesburg, 7 York Road, Parktown 2193, South Africa; mostafa.mabrouk@wits.ac.za (M.M.); yahya.choonara@wits.ac.za (Y.E.C.); pradeep.kumar@wits.ac.za (P.K.); lisa.dutoit@wits.ac.za (L.C.d.T.); 2 Refractories, Ceramics and Building Materials Department, National Research Centre, 33El Bohouth St. (former El-Tahrir St.), Dokki, Giza, P.O. 12622, Egypt

**Keywords:** poly(caprolactone), poly(acrylic acid), blend scaffold, bioactivity, ciprofloxacin drug delivery, silica microspheres

## Abstract

A new composite poly(caprolactone) (PCL) and poly(acrylic acid) (PAA) (PCL:PAA 1:5) scaffold was synthesized via dispersion of PCL particles into a PAA network. Silica microspheres (Si) (2–12 μm) were then prepared by a lyophilized micro-emulsion/sol-gel (Emugel) system using varying weight ratios. The model drug ciprofloxacin (CFX) was used for *in situ* incorporation into the scaffold. The physicochemical and thermal integrity, morphology and porosity of the system was analyzed by X-Ray Diffraction (XRD), Attenuated Total Refelctance Fourier Transform Infrared (ATR-FTIR), Differential Scanning Calorimetry (DSC), SEM, surface area analysis and liquid displacement, respectively. The mechanical properties of the scaffold were measured by textural analysis and *in vitro* bioactivity, biodegradation and pH variations were evaluated by XRD, FTIR and SEM after immersion in Simulated Body Fluid (SBF). The *in vitro* and *in vivo* studies of the prepared scaffold were considered as future aspects for this study. CFX release was determined in phosphate buffer saline (PBS) (pH 7.4; 37 °C). The incorporation of the Si microspheres and CFX into the scaffold was confirmed by XRD, FTIR, DSC and SEM, and the scaffold microstructure was dependent on the concentration of Si microspheres and the presence of CFX. The system displayed enhanced mechanical properties (4.5–14.73 MPa), *in vitro* bioactivity, biodegradation and controlled CFX release. Therefore, the PCL/PAA scaffolds loaded with Si microspheres and CFX with a porosity of up to 87% may be promising for bone tissue engineering.

## 1. Introduction

Poly(caprolactone) (PCL) is an aliphatic polyester that has been widely used in the biomedical field and for drug delivery applications due to its well-known properties such as biocompatibility, low immunogenicity, permeability and degradation products of low acidity compared with other aliphatic polyesters [1,2]. However, PCL is hydrophobic, has a relatively high crystallinity and is inflexible in terms of chemical interaction. This limits its use in biomedical applications such as for bone tissue engineering. Therefore, various techniques have been explored to overcome these challenges such as surface grafting and inorganic compositing of the PCL backbone. The complexation of PCL with other biocompatible polymers has also shown promise in expanding the applications of PCL in tissue engineering [3,4].

Polyanionic macromolecules such as poly(acrylic acid) (PAA) have also been broadly investigated as an organic substrate for the production of calcium carbonate particles via electrostatic interaction between the carboxyl groups and calcium ions in solution [5,6]. This introduces a new approach for organic–inorganic composite synthesis with superior performance as a biomaterial. However, PAA is not an ideal additive for PCL modification due to its aqueous swellability. The introduction of Silica (Si) microspheres with PAA as a polyanionic macromolecule may overcome this limitation. Si is a well-known matrix for microsphere preparation due to its chemical inertia, low density, high thermal and mechanical stability as well as ease of functionalization [7,8].

Therefore the focus of this study was to enhance the properties of both PCL and PAA through alteration of their properties by first preparing a new lyophilized PCL/PAA blended scaffold with superior mechanical stability and bioactivity for drug incorporation. In addition, a novel synthesis method for Si microspheres using a lyophilized micro-emulsion/sol-gel (Emugel) approach is reported. Si microspheres were prepared for inclusion into the PCL/PAA scaffold at concentrations of 10, 20 and 30 wt % to prepare the organic-inorganic composite scaffold. Ciprofloxacin (CFX) antibiotic was loaded as the model drug to control infection when the scaffold was potentially applied as an orthopedic implantable system for bone tissue engineering. The system was subsequently lyophilized to form a 3D scaffold and the physicochemical, thermal stability and physicomechanical properties were investigated by X-Ray Diffraction (XRD), Differential Scanning Calorimetry (DSC), Fourier Transform Infrared (FTIR) spectroscopy and Texture Analysis. The biodegradability and *in vitro* bioactivity of the new scaffold were examined by immersion of the scaffold in Simulated Body Fluid (SBF). *In vitro* drug release studies of the CFX-loaded scaffolds were undertaken in phosphate buffer saline (PBS) at 37 °C over 28 days.

## 2. Materials and Methods

### 2.1. Materials

Tetraethyl orthosilicate (TEOS) minimum assay 99% was purchased from Monitoring & Control Laboratories (Pty) Ltd., Johannesburg, South Africa, poly(caprolactone) (PCL) (*M*_W_ = 80,000 g/mol) and poly(acrylic acid) (PAA) (*M*_W_ = 450,000 g/mol) were purchased from Sigma-Aldrich Co., (Darmstadt, Germany). Polyethylene glycol (PEG) (*M*_W_ = 6000 g/mol) was purchased from Unilab Co., Johannesburg, South Africa. Dichloromethane (DCM) and hydrochloric acid 32% (HCL) (*M*_W_ = 36.46 g/mol) were purchased from (ACE Co., Johannesburg, South Africa). Ciprofloxacin (CFX) 98.0% (HPLC) (*M*_W_ = 331.34 g/mol) was purchased from Fluka, Neu-Ulm, Germany. The water used was Mille-Q water from a Millipore Water Purification System (Merck Chemicals GmbH, Darmstadt, Germany).

### 2.2. Synthesis of the Silica Microspheres

Silica microspheres were prepared using a novel micro-emulsion/sol-gel/lyophilization (EmuGel) method. Briefly, 1 mL of Tween^®^ 80 and 2 g of PEG were dissolved in 100 mL of isopropyl alcohol at 60 °C under continuous agitation until the solution was transparent. After, 20 mL of TEOS was added to the solution, and the pH was adjusted to a value of 2 using HCL to achieve silica hydrolysis. The mixture was then stirred for 30 min at 21 °C. Thereafter, 80 mL of deionized water was titrated on the mixture continues stirring for another 30 min at room temperature. The prepared solution was then incubated at 50 °C until a gel was formed. The gel was then washed several times with deionized water, frozen at −80 °C overnight and lyophilized at −64 °C over 24 h.

### 2.3. Preparation of the PCL/PAA Composite Scaffold

The PCL/PAA scaffold was synthesized by the dispersion of 5% *w*/*v* PCL prepared in DCM into 10% *w*/*v* PAA prepared in deionized water at 60 °C with a weight ratio (PCL:PAA = 1:5). In particular, PCL was dispersed in the PAA solution after cooling to 21 °C under continuous agitation for 30 min to allow the organic solvent (DCM) to evaporate. The silica microspheres were then added separately to the blends in varying wt % (10, 20 and 30 wt %) and agitated overnight using a magnetic stirrer in a closed system to maintain the *w*/*v* concentration. Ciprofloxacin was suspended in distilled water and *in situ* loaded with (10 wt %) into all the scaffolds continue stirring for 1 h till homogenous mixture was obtained. Thereafter, the blend was transferred into molds to form cylinders (D × H = 15 mm × 10 mm) and stored at −80 °C overnight before lyophilization (VIRTIS 2KBTXL-75 Benchtop SLC Freeze Dryer, SP Scientific, Suffolk, UK) for 24 h at −64 °C. The various scaffolds formulations processed are listed in Table 1.

### 2.4. Morphological and Microstructure Characterization of the PCL/PAA Scaffold

The micro-architecture of the scaffolds was assessed qualitatively using SEM and quantitatively via BET surface area measurements, as well as the liquid displacement method. SEM analysis was undertaken on the silica microspheres, CFX and PCL/PAA scaffolds. For the scaffolds, a thin sample of scaffold sheared from the center after immersion in liquid nitrogen for 2 min was used for analysis. Scaffold analysis was investigated using a Phenom^TM^ Benchtop SEM (FEI Company, Hillsboro, OR, USA). Samples were rendered electrically conductive before analysis through gold-sputter coating (SPI Module^TM^ Sputter Coater, SPI Supplies, West Chester, PA, USA) and were attached to the SEM stub using adhesive carbon tape. The BET surface area measurements were recorded utilizing a Porositometric Analyzer (Micromeritics ASAP 2020, Norcross, GA, USA). Briefly, 100 mg of scaffold underwent degassing and, thereafter, an absorption and desorption cumulative phase for accurate analysis of the surface area. Scaffolds were also analyzed by the liquid displacement method. Practically, each scaffold was submersed in paraffin oil for 1 h and centrifuged for 15 min at 5000 rpm, allowing the liquid to fill all pores. According to the liquid displacement theory the volume of a scaffold immersed in the fluid is equal to the volume of the displaced fluid, according to Mabrouk *et al.*, 2014 [9]. The porosity was calculated using Equation (1).

(1) $$P\%=\frac{\left(W_{1}-W_{3}\right)}{\left(W_{2}-W_{3}\right)}\times 100$$

where (*W*_1_) is the weight of the scaffold before immersion, (*W*_2_) is the weight of the scaffold after immersion and (*W*_3_) is the weight after drying, from which the porosity percentage (*P*%) can be obtained. All the experiment were undertaken in triplicate (*N* = 3).

### 2.5. Characterization of the Thermal Behavior of the PCL/PAA Scaffolds

The thermal behavior of the scaffolds before and after silica microspheres and CFX loading was determined by DSC (Mettler Toledo, DSC1, STARe System, Schwerzenback, Switzerland). With a view to evaluate the influence of silica microspheres and CFX on the thermal behavior of the prepared scaffolds by investigating the crystallization phenomenon. Samples (10 ± 1 mg) were placed into 40 µL aluminum pans and heated from 20 to 500 °C with a heating rate of 10 °C/min.

### 2.6. Physicochemical Integrity Analysis of PCL/PAA Scaffolds

In order to demonstrate the effect of loading silica microspheres, as well as CFX on the physicochemical integrity of the polymer blend scaffolds, XRD and FTIR analysis were undertaken. A Rigaku MiniFlex600 Benchtop X-ray Diffractometer (Rigaku Corporation, Tokyo, Japan) fitted with a 600 W X-ray generator, a counter monochromator to cut X-rays other than Cu Ka X-rays, and a high-intensity D/tex Ultra high speed 1D detector was used for the phase assessment of the PCL/PAA blind before and after Si microspheres and CFX loading with reference to the native polymers; PCL and PAA. ATR-FTIR spectra were recorded for all samples using a Perkin Elmer Spectrum 2000 FTIR spectrometer, employing a single-reflection diamond MIRTGS detector (PerkinElmer Spectrum 100, Llantrisant, Wales, UK). All samples were analyzed by a universal ATR polarization accessory for the FTIR spectrum series at a resolution of 4 cm^−1^. Samples were placed on a diamond crystal running each sample 100 times in order to reduce the signal-to-noise ratio to a minimum of 10, in the range of 4000–600 cm^−1^, using a constant pressure of 120 psi.

### 2.7. Determination of the Physicomechanical Properties of the PCL/PAA Scaffolds

The effect of silica microspheres as well as CFX on the physicomechanical properties of the prepared scaffolds were determined by Texture Analyzer. The compressive strength, rigidity gradient, deformation energy, and matrix resilience (%) were measured using a Texture Analyzer (TA.XT*plus* Stable Microsystems, Surrey, UK). A compressive stress was applied by the textural probe, according to the following conditions: a pre-test speed of 0.5 mm/s, a starting compression force of 1 kg, a distance of 5 mm (50% of the sample length), a test speed of 1 mm/s and an acquisition rate of 200 points/s. Additionally, the scaffolds were allowed to recover to their original dimensions.

### 2.8. *In Vitro* Bioactivity, Biodegradation and Micro-Environmental pH Variation Analysis

The degree of bioactivity of the PCL/PAA scaffold before and after loading silica microspheres and CFX was determined by submersing each scaffold in 50 mL of SBF (pH 7.4; 37 °C) at 7, 14, 21 and 28 days. The SBF was prepared according to Kokubo and co-workers 2006 [10]. Furthermore, the scaffolds were removed from SBF and washed with deionized water before analyzed by XRD, FTIR and SEM. The measurement of the degradation rate of the PCL/PAA scaffold was undertaken in SBF at the same conditions for the bioactivity experiment. At each time period, a scaffold sample was collected and washed several times in deionized water to ensure removal of the adsorbed ions and subsequently dried at 21 °C. According to Srinivasan and co-workers 2012 [11], the degradation of scaffolds was calculated using Equation (2). Three scaffolds from each sample were examined at each immersion time point. The micro-environmental pH variation in the SBF at different time points were measured with a pH meter in order to prognosticate the interaction mechanism between the silica microspheres and CFX-loaded scaffolds and the native PCL/PAA blend with the SBF constituents. The results are demonstrated as an average value ± standard deviation (*N* = 3).

(2) $$\text{Degradation}\%=\frac{\left(W_{0}-W_{t}\right)}{W_{0}}\times 100$$

where the scaffold weight before soaking in SBF was (*W*_0_) and the scaffold weight after specific soaking time was (*W*_t_).

### 2.9. *In Vitro* Analysis of Ciprofloxacin Release

To determine the release behavior of CFX form the PCL/PAA blend scaffold, *in vitro* release studies were undertaken in PBS (pH 7.4; 37 °C): 100 mL of PBS, previously heated at 37 °C, was added to glass vessels maintained at 37 °C within a shaking incubator (50 oscillations·min^−1^). At predetermined time intervals, 1 mL samples of the release medium were withdrawn and the CFX concentration was determined spectrophotometrically at 277 nm using UV-Vis spectroscopy (Lambda 25 UV/Vis Spectrophotometer, PerkinElmer, Waltham, MA, USA). Before analysis, the samples were filtered and diluted with PBS. The withdrawn samples were replaced with drug-free PBS in order to maintain sink conditions. CFX release was monitored over 28 days and all experiments were performed in triplicate (*N* = 3). CFX is well known to be highly effective against Gram positive and negative bacteria, therefore, an antibacterial test was not necessary.

## 3. Results and Discussion

### 3.1. Morphological and Microstructure Property Assessment

**Figure 1:**
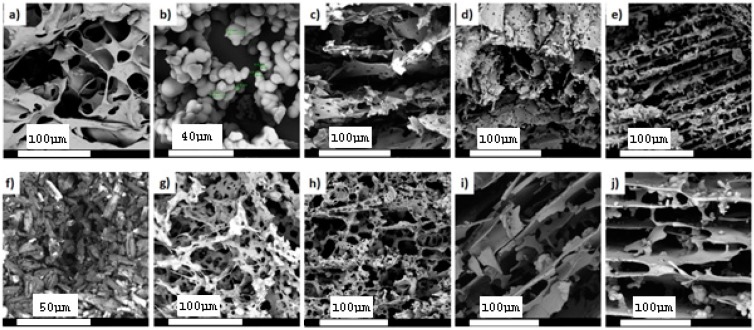
SEM images before immersion in Simulated Body Fluid (SBF) of (**a**) PCL/PAA blend; (**b**) silica microspheres; (**c**) PCL/PAA/S1 blend; (**d**) PCL/PAA/S2 blend; (**e**) PCL/PAA/S3 blend; (**f**) CFX; (**g**) PCL/PAA/CFX blend; (**h**) PCL/PAA/S1/CFX blend; (**i**) PCL/PAA/S2/CFX blend, and; (**j**) PCL/PAA/S3/CFX blend.

Due to the importance of inorganic phase incorporation into the PCL/PAA blend matrix on the final microstructure properties of the scaffolds synthesized via lyophilization [9,10,11,12,13,14,15,16], the influence of different silica microspheres ratios on the surface area and the porosity of the blend scaffolds were analyzed. Figure 1 shows the SEM images of the scaffolds before and after the loading of silica microspheres and CFX, as well as pure silica (Figure 1b) microspheres and CFX (Figure 1f). The PCL/PAA blend scaffold exhibited a non-uniform interconnected porous microstructure with smooth pore walls. Upon the silica microspheres loading into the PCL/PAA blend scaffold, the pores realigned into a uniform microstructure (Figure 1c). This result could be explained by the incorporation of an inorganic phase (silica microspheres) that facilitated the realignment of the PCL/PAA scaffold microstructure. Furthermore, an increase in uniformity was linear with an increase of the silica microspheres percentage as shown in Figure 1d,e. In addition, the presence of CFX, specifically with silica microspheres, loaded scaffolds increased the pore volume as shown in Figure 1i,j which is favorable for tissue engineering applications. The porosity and surface area decreased, as listed in Table 2. These results were due to the presence of the silica microspheres that restricted the number of open pores. However, the silica microspheres exhibited particle size (2–12 µm) which in turn increased the total surface area of the scaffolds. A similar result was reported previously for other silica microspheres [17,18]. Furthermore, it is worthy to note that the higher surface area supports better cell attachment than a lower surface area scaffold microstructure.

### 3.2. Thermal Behavior of the PCL/PAA Scaffolds

**Figure 2:**
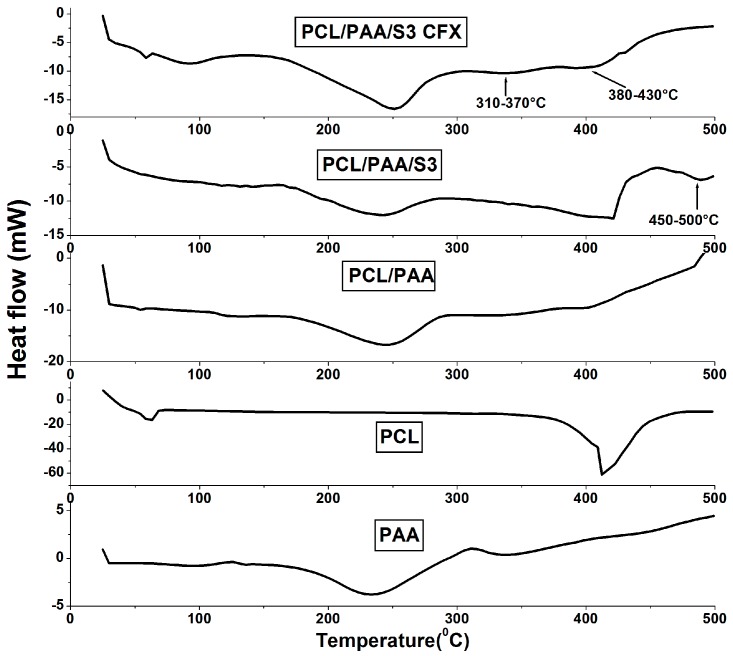
Thermograms of the PCL/PAA blend before and after loading of silica microspheres and CFX with respect to the native polymers, PCL and PAA.

Figure 2 shows the thermal behavior of the scaffolds before and after the individual loading of silica microspheres and CFX. The thermogram of all scaffolds exhibited identical thermal behavior of native PAA, confirming its domination except for the endothermic broad peak at 380–430 °C. This peak was attributed to the presence of the PCL. Silica microspheres-loaded scaffolds had an identical thermal pattern with increased intensity for the PCL peak. This result was due to the overlapping of the PCL peak with the *T*_g_ peak of silica microspheres. The silica microspheres *T*_g_ peak was recorded at 450–550 °C as reported for silicate glass [12]. The exothermic peak observed at 310–370 °C corresponded to the new intermolecular hydrogen bonds that occurred between the PCL/PAA blend and the CFX for scaffolds loaded with CFX [13]. The DSC results confirmed the domination of PAA and the successful entrapment of both silica microspheres and CFX into the PCL/PAA blend with the thermal integrity of the polymer backbones maintained.

### 3.3. Assessment of the Physicochemical Structure Stability

**Figure 3:**
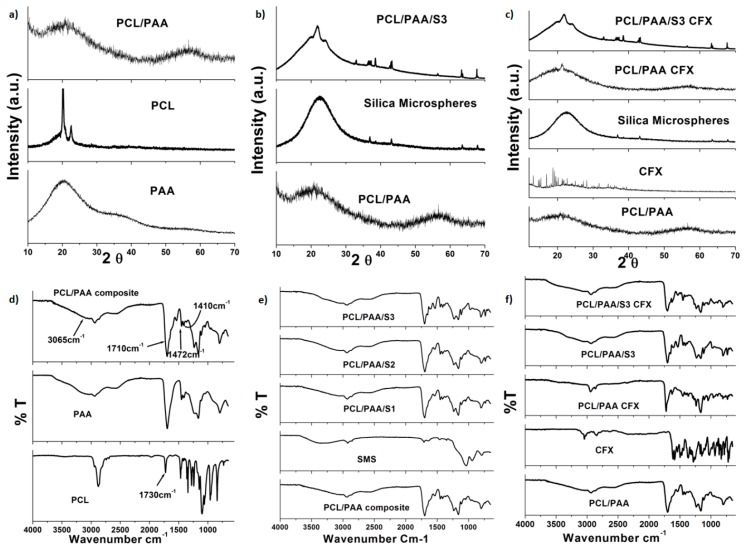
X-ray diffraction (XRD) patterns of (**a**) blending of PCL with PAA; (**b**) incorporation of silica microspheres into PCL/PAA blends; (**c**) encapsulation of CFX into the PCL/PAA blends and Fourier Transform Infrared (FTIR) spectra of; (**d**) blending of PCL with PAA; (**e**) incorporation of silica microspheres into PCL/PAA blends; (**f**) encapsulation of CFX into the PCL/PAA blends.

The XRD pattern of PAA showed no presence of any crystalline phases, being completely amorphous, while PCL exhibited some diffraction peaks. Hence, it has been identified as a semi-crystalline structure due to the superior concentration of esters groups. The PCL/PAA scaffold demonstrated an amorphous XRD pattern which is almost the same as PAA pattern, as illustrated in Figure 3a. This result confirmed the homogenous dispersion of PCL into the PAA network. Moreover, the loading of Si microspheres and CFX was confirmed by the XRD patterns of PCL/PAA/S3 and PCL/PAA/S3/CFX blends presented in Figure 3b,c. These curves directly verify the summary of contributions from PCL/PAA amorphous phase with Si microspheres, as well as CFX semi-crystalline structure, similar results were early reported [9,12]. The FTIR spectra of the scaffolds are shown in Figure 3d–f. PCL and PAA have counter solubility organic and non-organic soluble polymers. Thus, the PCL/PAA scaffold FTIR spectrum exhibited an identical spectrum to PAA, confirming its domination. In particular, the observed bands for the PCL/PAA scaffold were also observed for PAA. These included a strong band at 1710 cm^−1^ corresponding to the vibration stretching of carboxylic acid groups C=O, an O–H stretching at 3065 cm^−1^, the characteristic asymmetrical COO− band at 1410 cm^−1^ and the symmetrical COO− band at 1472 cm^−1^ [14]. Moreover, the intensity of the main characteristic band of PCL observed at 1730 cm^−1^ corresponded to carbonyl stretching [1,3,4], which was increased and broadened due to overlapping with PAA bands, specifically at 1550–1750 cm^−1^. The silica microspheres-loaded PCL/PAA scaffolds (Figure 3b) showed identical bands to the native PCL/PAA blend, which indicated that there was no interaction. In addition, CFX-loaded PCL/PAA blend scaffolds (Figure 3c) also revealed all bands of the native PCL/PAA blend, suggesting that there was no interaction between PCL/PAA, silica microspheres and CFX. These results were consistent with results reported by Perumal and co-workers [15] and with the DSC results obtained in this study.

### 3.4. Assessment of the Mechanical Properties of the Scaffold

Undoubtedly, the optimization of the mechanical properties such as the Compressive Strength (CS), Rigidity Gradient (RG), Deformation Energy (DE), and Matrix Resilience (MR) of the scaffolds is one of the most challenging aspects to biomaterials scientists. The mechanical properties of scaffolds directly interfered with the cell-scaffold interaction and also determined the appropriate site of implantation [19]. The Rigidity Gradient refers specifically to the scaffold unit migration guided by gradients in substrate rigidity. The Deformation Energy is defined as the absorbed energy that causes deformation of the elastic scaffold. The Matrix Resilience (%) is defined as the competence of a scaffold to release energy while under elastically deformation, and release that energy after unloading [20].

**Figure 4:**
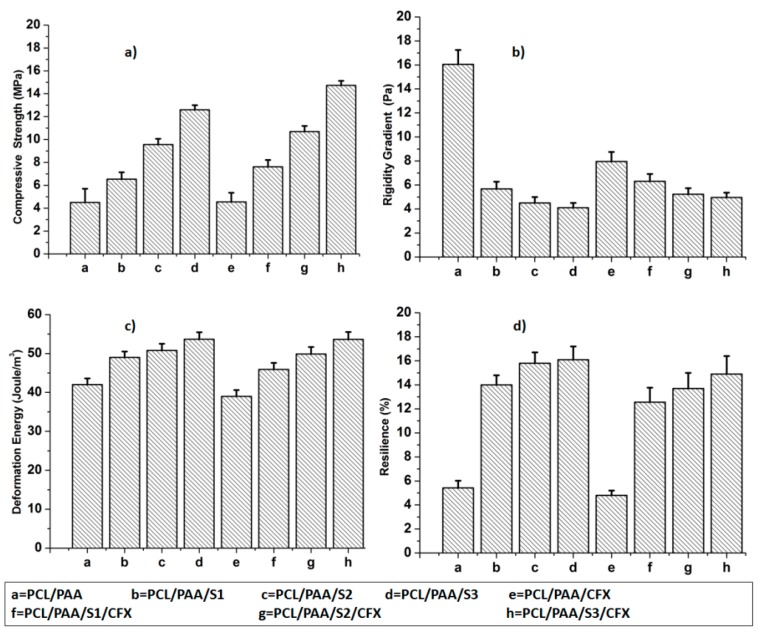
Physicomechanical properties of the prepared scaffolds represented by (**a**) Compressive Strength; (**b**) Rigidity Gradient; (**c**) Deformation Energy and; (**d**) Matrix Resilience (%).

Figure 4 represents the mechanical properties of the PCL/PAA scaffolds before and after silica microspheres and CFX loading with reference to the native PCL/PAA scaffold. The CS, DE and MR (%) of all the investigated scaffolds increased compared with the native PCL/PAA blend scaffold, as shown in Figure 4a,c,d. In particular, the CS, DE and MR (%) increased from 4.5 ± 0.9 MPa, 42.0 ± 0.2 Joule/m^3^ and 5.42% ± 1.2% (PCL/PAA scaffold) to 14.73 ± 1.1 MPa, 53.65 ± 0.4 Joule/m^3^ and 14.90% ± 1.2% (PCL/PAA/S3/CFX scaffold), respectively. In contrast, the RG of the silica microspheres-loaded scaffolds decreased compared with the native PCL/PAA scaffold, as shown in Figure 4b. These results are attributed to the clear porosity (%) decrement of the silica microspheres-loaded blend scaffolds and supported by the fact that silica microspheres are considered as inorganic filler [9,21]. In fact, such a decrease has not been recognized for PCL/PAA blend scaffolds since the structure is initially porous (Figure 1a). Implanted scaffolds in load-bearing bone should possess properties of adequate porosity and mechanical properties to bear the applied *in vivo* load and to prevent new tissue damage [22]. The CS for the silica microspheres and CFX loaded scaffolds of ˃14 Mpa is applicable for bone tissue engineering. However, a range of values is typically considered appropriate due to differences in cortical and trabecular bone structure [23].

### 3.5. In Vitro Bioactivity, Biodegradation and SBF pH Variations

#### 3.5.1. *In Vitro* Bioactivity

**Figure 5:**
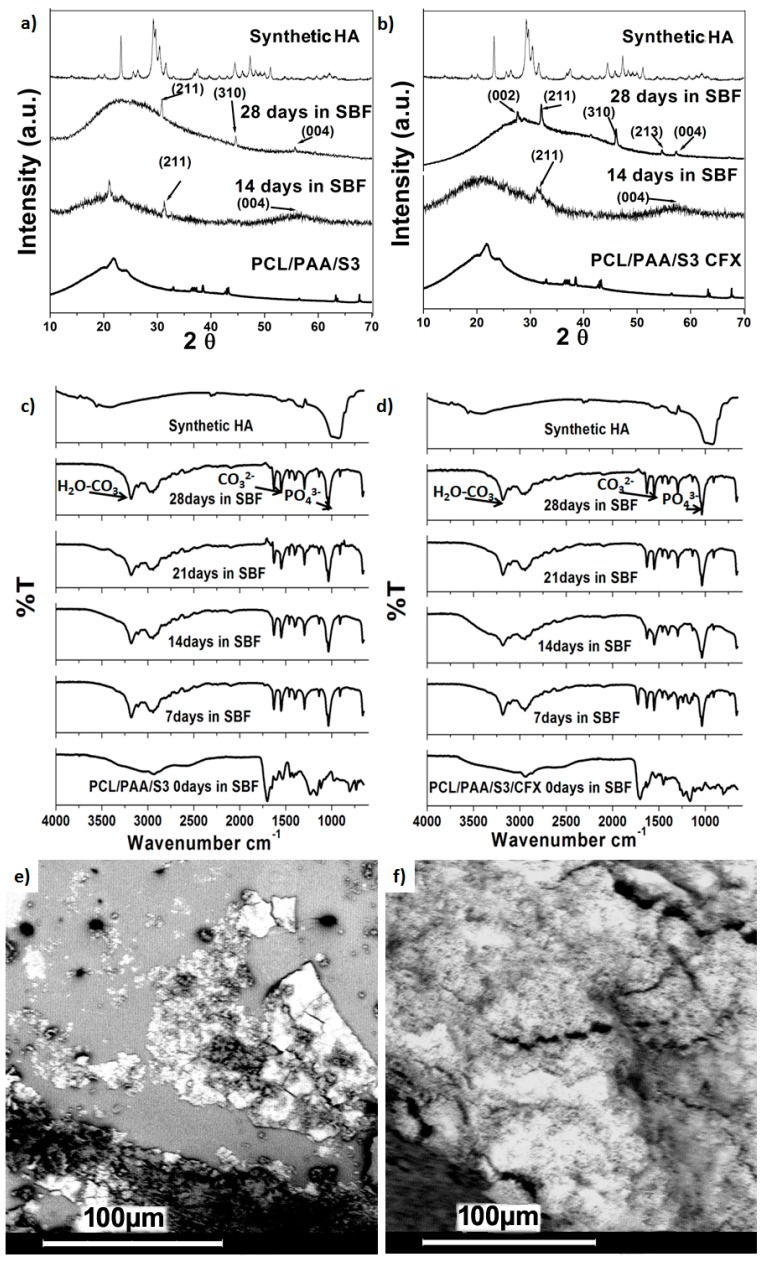
XRD patterns before and after immersion in SBF of (**a**) PCL/PAA/S3 blend and (**b**) PCL/PAA/S3/CFX blend; FTIR spectra before and after immersion in SBF of (**c**) PCL/PAA/S3 blend and (**d**) PCL/PAA/S3/CFX blend; SEM images before and after immersion in SBF of (**e**) PCL/PAA/S3 blend and (**f**) PCL/PAA/S3/CFX blend.

The *in vitro* bioactivity of the scaffolds was assessed by means of XRD, FTIR and SEM after immersion of scaffolds in SBF for varying time intervals. XRD analysis and FTIR spectra before and after immersion in SBF compared with synthetic hydroxyapatite (HA) are represented in Figure 5a–d. Based on the physicochemical and physicomechanical studies, PCL/PAA/S3 and PCL/PAA/S3/CFX scaffolds were selected to be investigated after immersion in SBF. These scaffolds showed a remarkable change after immersion in SBF due to degradation of the PCL/PAA blends scaffolds. However, the degradation products of PCL and PAA are a naturally occurring metabolite in the human body [1,24]. The XRD pattern for the Si-loaded scaffold as well as CFX-loaded scaffold showed amorphous calcium phosphate precipitations after 14 days of immersion in SBF, as confirmed by the presence of the following peaks that were observed at 32° (211), and 53° (004). After 28 days of immersion in SBF, the calcium phosphate layers precipitated on the surface of these scaffolds were more crystallized, as affirmed by the following peaks that were observed at 32° (211), 40° (310), 50° (213) and 53° (004), as earlier reported for organic scaffold loaded with in organic filler [25,26]. However, the PCL/PAA/S3/CFX scaffold showed relatively higher bioactivity than the PCL/PAA/S3/scaffold as confirmed by presence of peak at 28° (002),which is attributed for HA.

Further, the observed strong vibrational band after immersion in SBF at 1020 cm^−1^ was ascribed to PO_4_^3−^ groups that suggested the formation of amorphous calcium phosphate (ACP), which clearly supports the formation of an apatite crystalline layer over the surface of the scaffolds [27]. Thus, the presence of bands due to PO_4_^3−^ groups at 1020 cm^−1^ in the FTIR spectra after immersion indicated the formation of a HA layer on the surface of the PCL/PAA scaffolds. Moreover, the presence of the band at 1540 cm^−1^ due to a CO_3_^2−^ functional group and the band around 3000–3200 cm^−1^ was attributed to the bridging mode of H_2_O-CO_3_, which signified the incorporation of carbonate anions from the SBF into the apatite crystal lattice [28]. The accurate analysis of the FTIR spectra of immersed scaffolds revealed that the intensity of bands due to phosphate and carbonate groups gradually increased with increase in the silica microspheres content up to 30 mol %, indicating a great degree of bioactivity for the PCL/PAA/S3/CFX scaffold. SEM images of the under investigated scaffolds after immersion in SBF over 28 days are reported in Figure 5e,f. The morphology of the CFX-loaded blend scaffolds showed relatively high bioactivity rate compared to scaffolds that did not contain CFX. This result is consistent with the XRD and FTIR results after immersion in SBF and could be explained due to the presence of CFX that was approved as an enhancer for bone-like apatite formation [9,13,23]. The *in vitro* cell culturing and *in vivo* studies of the prepared scaffold were considered as future aspects for this study.

#### 3.5.2. Biodegradation and SBF pH Variations

**Figure 6:**
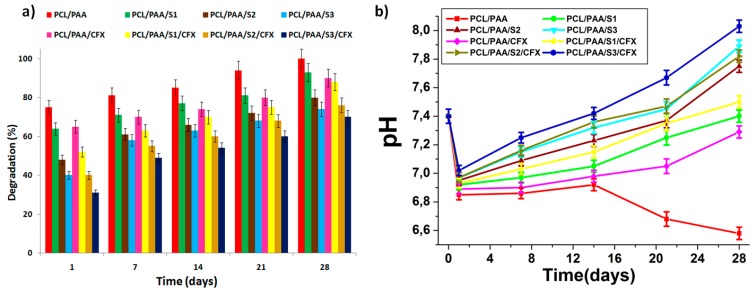
(**a**) Biodegradation (%) and (**b**) SBF pH variations for all the prepared scaffolds after different interval times of immersion in SBF.

The biodegradation and pH measurements were conducted over a period of 28 days while the scaffolds were incubated in SBF under the same conditions of the biomineralization experiment. Figure 6 represents the biodegradation (%) and the SBF pH variations of the investigated scaffolds. A significant decrease in SBF pH values was observed at the initial stage after immersing the scaffolds in SBF. This phenomenon was caused by the direct degradation of the PCL/PAA scaffolds to PCL and PAA acidic degradation products, as reported previously [29,30]. This decrease occurs after 2 days of immersion in SBF. In particular, after 2–7 days, a noticeable decrease in pH (from 7.4 to 6.8) was observed, combined with a rapid scaffold biodegradation rate (35%–75%). It is highlighted that the biodegradation rate of the silica microspheres and CFX-loaded scaffolds were slower than the silica microspheres and CFX-free scaffolds. These results are obtained due to the fact that released silica microspheres and CFX stabilized and compensated for the effect of the PCL/PAA acidic degradation products, which in turn stabilized SBF pH.

Furthermore, from days 7–28, a slower biodegradation rate was observed for all scaffolds (25%–45%) combined with a pH increase (from 6.8 to 8.0), except for the PCL/PAA scaffolds that showed significant pH decrease (from 6.8 to 6.5). This was well suited for carbonated apatite precipitation on the surface of the scaffolds [30]. Based on the bioactivity, biodegradation and SBF pH variations results, it can be deduced that higher quantities of silica microspheres (up to 30 mol %) was beneficial in order to stabilize and compensate for the effect of the PCL/PAA acidic degradation products.

The observed biodegradation behavior is attributed to both the PAA and silica microspheres within the scaffolds. It is well known that the rate of PCL degradation may be negligible compared to the PAA degradation rate and, therefore, does not play a significant role in the biodegradation in SBF after 28 days of immersion, while PAA as a polyanionic macromolecule that is distinguished by promoting solubility in physiological fluids [31,32,33,34]. It is also worthy to note that when polyanionic macromolecules are blended with PCL, the hydrophilicity of PCL may be enhanced as a consequence of the high hydrophilicity and solubility of PAA. This realization was confirmed by the analysis of the biodegradation rate and SBF pH variation profiles 7–28 days after immersion in SBF.

### 3.6. Assessment of the Ciprofloxacin Release Behavior from the PCL/PAA Scaffolds

**Figure 7:**
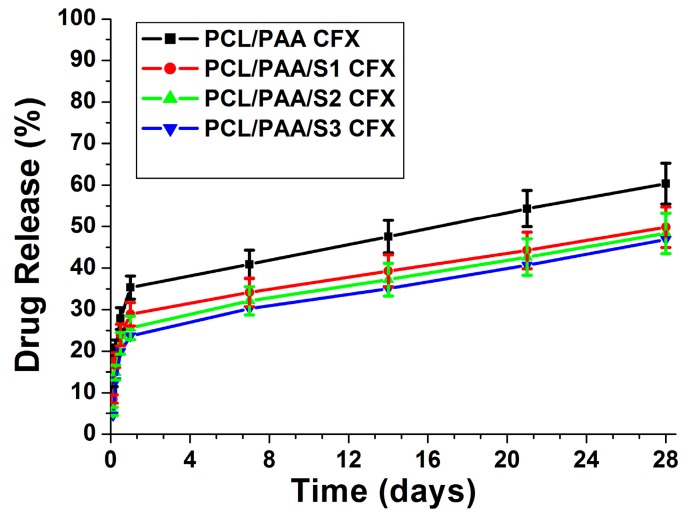
CFX release (%) from the silica microspheres loaded scaffolds after variant interval times of immersion in phosphate buffer saline (PBS) with reference to the silica microspheres free scaffold.

In order to investigate the effect of silica microspheres loading on the CFX release behavior, *in vitro* drug release studies were conducted for all CFX-loaded scaffolds in PBS, as shown in Figure 7. Generally, all scaffolds released 30% of CFX after 1 day and, thereafter, the release pattern followed linear behavior up to Day 28. The CFX release from both silica microspheres-loaded and silica microspheres-free scaffolds was relatively identical, except that the initial burst phase from the silica microspheres-loaded scaffolds was slightly reduced (20%), and the corresponding release rates were clearly down-regulated by the presence of silica microspheres. These results are due to the fact that CFX was incorporated into the PCL/PAA scaffold matrix through physical blending, as confirmed by SEM images Figure 1g–j. Therefore, the CFX was superficially adsorbed onto the silica microspheres-free scaffold matrices. Once exposed to PBS media, the physical attachment between CFX and the scaffold matrix was easily disentangled due to the hydrophilicity of both PAA and CFX, leading to a significant initial release phase, followed by linear behavior up to Day 28. In contrast, the presence of silica microspheres formed hydrogen bonding with CFX, which relatively decreased the burst effect of CFX from the silica microspheres-loaded scaffolds. These results were consistent with earlier reported results for sustained drug release from multi-phase blend scaffolds [35,36,37,38,39]. In summary, the higher quantities of silica microspheres regulated the release profiles of CFX from the scaffolds. Based on the results illustrated in Figure 7, and correlating this with the microstructure properties of the scaffolds, it can be deduced that higher silica microspheres concentrations (30 mol %) are recommended for a sustained and prolonged CFX release due to their relatively low porosity compared with the low silica microspheres concentrations (0 and 10 mol %).

## 4. Conclusions

The present study supports the development of a novel biodegradable lyophilized PCL/PAA scaffold via the dispersion of PCL into a PAA network. Silica microspheres were successfully prepared for the first time by a micro-emulsion/sol-gel/lyophilization method with particle sizes ranging between 2–12 µm. The silica microspheres-loaded PCL/PAA scaffolds exhibited an enhanced microstructure as well as desirable mechanical properties, bioactivity, biodegradation and CFX release behavior compared with silica microspheres-free scaffolds. More to the point, it was confirmed that the biodegradation rate and bioactivity of the scaffolds were strongly affected by the presence of silica microspheres. The results recommend the potential use of the PCL/PAA scaffolds for bone tissue engineering. The *in vitro* cell culturing and *in vivo* studies of the prepared scaffold were considered as future aspects for this study.

## Figures and Tables

**Table 1:** Formulation template of silica microspheres and ciprofloxacin-loaded scaffolds. PCL: poly(caprolactone); PAA: poly(acrylic acid); CFX: ciprofloxacin.

Sample	PCL/PAA (wt %)	Silica microspheres (wt %)	CFX (wt %)
PCL/PAA	100	-	-
PCL/PAA/S1	90	10	-
PCL/PAA/S2	80	20	-
PCL/PAA/S3	70	30	-
PCL/PAA/CFX	90	-	10
PCL/PAA/S1/CFX	80	10	10
PCL/PAA/S2/CFX	70	20	10
PCL/PAA/S3/CFX	60	30	10

**Table 2:** BET surface area and porosity (%) results by liquid displacement of all scaffold blends.

Sample	BET Surface area	Porosity (%) by liquid displacement method
PCL/PAA	3.87± 0.108 m^2^/g	87.10 ± 2.50
PCL/PAA/S1	19.31 ± 0.20 m^2^/g	81.35 ± 2.18
PCL/PAA/S2	26.51 ± 0.25 m^2^/g	77.45 ± 1.90
PCL/PAA/S3	45.37 ± 0.39 m^2^/g	51.74 ± 1.68
PCL/PAA/CFX	3.99 ± 0.07 m^2^/g	81.83 ± 2.70
PCL/PAA/S1/CFX	6.28 ± 0.11 m^2^/g	76.13 ± 2.11
PCL/PAA/S2/CFX	9.87 ± 0.19 m^2^/g	63.10 ± 1.87
PCL/PAA/S3/CFX	25.92 ± 0.33 m^2^/g	58.06 ± 1.63
